# Bringing nuclear materials discovery and qualification into the 21^st^ century

**DOI:** 10.1038/s41467-020-16406-2

**Published:** 2020-05-22

**Authors:** Jeffery A. Aguiar, Andrea M. Jokisaari, Matthew Kerr, R. Allen Roach

**Affiliations:** Idaho National Laboratory, 2525 Fremont Ave., Idaho Falls, ID 83415 USA

**Keywords:** Metals and alloys, Nuclear physics

## Abstract

Time horizons for nuclear materials development and qualification must be shortened to realize future nuclear energy concepts. Inspired by the Materials Genome Initiative, we present an integrated approach to materials discovery and qualification to insert new materials into service.

## Nuclear materials, past and present

The world’s energy needs have grown alongside the rising population and are now greater than ever. Part of this growing energy demand is the electrification and modernization of aging infrastructure through clean, secure, and safe sources of energy^1^. Nuclear power can achieve these energy needs while meeting these high safety standards and, in support of these objectives, the U.S. Congress has mandated demonstrations of advanced reactor technology by 2030^2^. Many advanced designs present more corrosive, hotter, and higher-irradiation environments than those in today’s nuclear reactor fleet, meaning potential new materials and processes must be developed in a timely manner. The final and crucial step of materials development is qualification, where it is determined that a fabricated part has the desired properties and that we know how these properties will evolve in the service environment.

While the timescale for materials design in other industries has been shortened to as low as three years^3^, nuclear materials development takes considerably longer and still relies on a largely iterative design cycle because of the need for irradiation experiments to understand in-reactor performance^4^. Irradiation profoundly impacts the evolution of material properties and this presents a significant barrier to qualifying new materials, as qualification remains based on insights into irradiation effects from historic large-scale qualification experiments and service experience with known materials^5^. This limited understanding of irradiation effects on materials has meant that efforts to successfully qualify and deploy innovative nuclear fuels and materials (e.g., TRISO fuels and advanced zirconium cladding alloys) take several sequential irradiation campaigns to complete, with each cycle lasting roughly a decade. However, there are a growing number of fuel system cladding concepts that have been developed in a single cycle (e.g., coated and iron-based claddings). This suggests change is possible with the adoption of a new development paradigm.

## Taking a more modern approach

Generally, to develop new materials for nuclear energy applications, environmental and irradiation conditions must be correlated to materials’ evolution and degradation in service. Novel structural materials must mitigate embrittlement mechanisms and reduce void swelling and creep. Fuel cladding, which protects the fuel, must maintain structural integrity during normal operating conditions and accident scenarios. The fuel itself must not release fission products, must meet stringent thermal properties requirements, and must not chemically react the cladding and coolant. This effort requires strategic design and development of advanced materials, such as the creation of microstructures more resistant to irradiation by harnessing the properties of specific material defects.

Since materials remain in service for a number of years, effective irradiation modeling for qualification and performance prediction is just as critical to shorten the development timeline. An irradiation damage event occurs on the scale of picoseconds, resulting in a population of radiation-induced defects that remain after the damage event and evolve on timescales ranging from seconds to years. These defect populations may or may not reach a steady state, and further damage events can interact with those same defects. Bridging these time scales computationally will reduce the reliance on irradiation testing.

Modern experimental and modeling techniques including data analytics, high-throughput experiments, and machine learning, can be leveraged to provide these rapid advancements in nuclear energy and materials^6^. One way this is being achieved is through the Nuclear Materials Discovery and Qualification initiative (NMDQi), a comprehensive program spanning national laboratories, academia, and industry.

NMDQi was launched in 2020 led by Idaho National Laboratory to address the need for development of new nuclear materials on shorter timelines. As a national effort, NMDQi will demonstrate processes and test platforms for accelerated nuclear materials fuel development within the next 5 years, and qualification within the next 10 years.

NMDQi is broadly patterned after the Materials Genome Initiative (MGI)^7^, which seeks to overcome the time-intensive, costly, iterative processes of traditional, non-nuclear materials development. The MGI created the Materials Innovation Infrastructure (MII), a framework that relies on a tightly integrated iteration of computational and experimental tools, allowing for rapid prediction of materials’ properties and performance, and ultimately, the ability to design materials concurrently with the design of the product^8^. Unfortunately, the existing MII and available materials design tools often depend on large amounts of experimental and computational data, which are not usually available for nuclear materials.

## The future of nuclear materials development and qualification

Irradiation effects represent an especially data-poor problem. While prior and ongoing work in post-irradiation examination (PIE), in-pile testing, and radiography are valuable resources in addressing the issue of data scarcity, the tools and capabilities to leverage these rich and diverse datasets have yet to be fully realized under current programs. The introduction of NMDQi aims to bridge this gap, since it focuses on generating tools and capabilities that integrate experimental and computational techniques to allow materials to be selected prior to fabrication. It will also provide crucial data to improve upon modeling. Specific ways in which it will achieve these goals are listed below:

### Physics-based, multiscale modeling and simulation

- High-throughput physics-based modeling, which makes predictions based on physical laws, can be combined with data analytics to infer trends and correlations. Doing so can create predictions of materials performance under irradiation at both short and long timescales, bridge results across length scales, and perform advanced image analyses^9,10^. Amongst other needs, this means developing accurate atomistic and mesoscale methods for predicting the effects of irradiation, temperature, stress, chemistry, and other physics on fuels and structural materials.

### Testing and characterization of nuclear materials

- Characterizing irradiated samples is time-consuming because of the nature of neutron irradiation and the difficulty of handling irradiated specimens. Highly irradiated specimens can damage sensitive electronic components, precluding the loading of multiple specimens at once. Furthermore, heavy shielding and mechanical manipulators are necessary for researchers’ safety, but they make using equipment, such as microscopes or mechanical testers, slow and difficult. Thus, the focus is not on the speed of sample cycling, but on what can be learned from a single sample via a single characterization method.

### Integration of data analytics and analysis

- Machine and deep learning methods must manage the challenge of data scarcity in the process of aiding in the discovery and development of new materials. Models must simultaneously be tightly integrated with current nuclear physics-based modeling, to provide predictive capabilities with physical meaning. While there are numerous successes with using physics-based modeling to understand performance, there are fewer examples that integrate these fundamentally informed models to infer performance of new materials: this will be a focus of NMDQi. In addition, NMDQi will define standards for data collection to ensure consistent utilization throughout the scientific community and create a common platform in order to link disparate databases.

## Needed nuclear materials advances

Several examples of materials-related areas to explore and improve upon include:

### Reactor pressure vessel (RPV) steels

- Neutron irradiation can limit the service life of RPV steels by changing its microstructure via, for example, radiation-induced segregation of alloying or trace elements, resolution of precipitates, and the formation of voids and dislocations. These notably affect the ductile-to-brittle transition temperatures, fracture toughness, and mechanical properties of the vessels. We focus on RPV steels because they provide a rich nuclear materials dataset resulting from decades of research on light water reactors. This dataset has yielded a fundamental understanding of the embrittlement phenomena involved at a range of length scales. By using the NMDQi approach and combining data analytics with physics-based modeling for this data set, we aim to advance models for property prediction and materials qualification^11^.

### Radiation and oxidation-resistant claddings

- Zirconium alloys have long served as fuel cladding material^12^ and they have been under continuous development since their introduction. However, they do not exhibit the physical and chemical stability necessary for advanced reactor conditions (e.g., between 700 °C and 1000 °C^13^). Novel high-temperature alloys, such as high-temperature ferritic steels and multiple principal element alloys (MPEAs), are being considered as an option, but the compositional space for MPEAs encompasses over a billion different potential alloys^14^, clearly indicating the need for a new method of materials selection.

### Advanced fuels

- Accident tolerant fuels will not only improve the safety of the current nuclear fleet, they will also improve fuel lifetimes and limit waste. Novel fuels must meet multiple performance metrics, such as neutron transparency, compatibility with cladding and coolant materials, and the ability to manage fission products. Modern accelerated irradiation campaigns, such as FAST and miniFuel, are further now capable of generating large amounts of information in relatively short periods that exceed the capacity of existing PIE facilities. NMDQi represents an opportunity to invest in high-throughput characterization facilities to support new approaches such as these for fuel qualification^4^.
